# Is kratom contributing to deaths in polypharmacy cases? A scoping review

**DOI:** 10.3389/ftox.2026.1970706

**Published:** 2026-10-26

**Authors:** Ella Matischak, Lyle D. Burgoon

**Affiliations:** 1 Raptor Pharm & Tox, Ltd., Apex, NC, United States; 2 Science for Healthy Families, Apex, NC, United States

**Keywords:** 7-hydroxymitragynine, 7-OH, kratom, mitragynine, polypharmacy

## Abstract

Kratom (*Mitragyna speciosa*) is used by an estimated 10–16 million Americans for self-management of pain, anxiety, and opioid use disorder, yet its causal contribution to associated deaths remains unresolved. This scoping review maps the available evidence on kratom’s role in polypharmacy-associated adverse outcomes, characterizes the pharmacological mechanisms through which kratom may contribute to harm in a co-substance context, identifies forensic and methodological barriers to causal attribution, and distinguishes the risk profile of traditional kratom from emerging semisynthetic 7-hydroxymitragynine (7- OH) products. A systematic literature search was conducted through April 2026 using PubMed Advanced Search Builder across fourteen discrete queries spanning pharmacological, epidemiological, clinical, and regulatory domains, supplemented by citation tracking and targeted grey literature review. Polysubstance use is the dominant pattern in kratom-associated fatalities across five independent data sources, with polydrug rates around 80%. Two pharmacologically coherent mechanisms support a plausible contributory role for kratom in polypharmacy deaths: additive and synergistic central nervous system (CNS) depression via μ-opioid receptor overlap and CYP enzyme-mediated pharmacokinetic amplification of co-substance toxicity. Concentrated semisynthetic 7-OH products, commercially emergent since 2023, produce substantially greater exposure to a potent MOP agonist than traditional kratom preparations, creating a distinct pharmacological hazard profile; however, their comparative polypharmacy fatality risk has not been established. Six fundamental data gaps prevent causal confirmation and limit evidence-based regulatory response. Closing them requires coordinated federal investment in forensic, pharmacokinetic, pharmacogenomic, and surveillance research.

## Introduction

Walking into most gas stations in the United States you can purchase a product that possesses pharmacological characteristics of potent opioids with no prescription and no requirement that the label disclose its opioid character. The product will likely be marketed with wellness claims including “natural, botanical, organic”, and will sit alongside energy drinks and protein bars with no indication that its primary active constituent binds the same receptor as fentanyl. If something goes wrong, the emergency physician who treats the overdose will likely run a standard immunoassay toxicology panel that will not detect it. The medical examiner who performs the autopsy may not test for it at all. And the federal agencies with authority to remove it from shelves have not done so, despite more than a decade of accumulating adverse event data, and a 1200% increase in poison center reports over the past decade (Eleanor Blair Towers et al., 2026).

That product is kratom.

Kratom (*Mitragyna speciosa*), a tropical tree indigenous to Southeast Asia, has been used for centuries in traditional medicine for its stimulant and analgesic properties. In recent decades, it has gained substantial popularity in the United States, where it is marketed and sold as a dietary supplement for the self-management of chronic pain, anxiety, depression, and opioid use disorder (OUD). According to the US Substance Abuse and Mental Health Services Association, approximately 5.28 million Americans aged 18+ years used kratom in 2024 (https://www.samhsa.gov/data/sites/default/files/reports/rpt56484/NSDUHDetailedTabs2024/NSDUHDetailedTabs2024/2024-nsduh-detailed-tables-sect8pe.htm?s=kratom&). The product is now widely available across retail channels, from gas stations to smoke shops to online marketplaces, with surveys documenting kratom in more than 62% of California vape shops and specialty tobacco retailers (Bowdring et al., 2023). Ready-to-drink kratom beverages have emerged as a rapidly growing product category, with 107 such products identified in a 2025 market analysis, sold across convenience stores, grocery stores, and online platforms (Perry and Chin, 2025).

Adverse event surveillance data reflect this growth. Analysis of the FDA Adverse Event Reporting System (FAERS) identified 489 deduplicated kratom-related adverse drug reaction reports between 2004 and 2021, with 94.2% of those reports filed after 2018. This pattern is more consistent with increased awareness and surveillance than with a sudden spike in adverse outcomes (Li et al., 2023). Among the signals identified, accidental death showed a reporting ratio 63.1-fold greater than would be expected by chance. Eight additional signals were identified for addiction and withdrawal, and an excess seizure signal was also detected (Li et al., 2023). The FAERS system is passive, voluntary, unverified, and disproportionate reporting ratios cannot establish that kratom caused any individual adverse outcome. They do, however, establish that kratom use is associated with serious harm at a scale that warrants systematic scientific investigation. More recent surveillance data from the National Poison Data System documents a 1,200% increase in kratom-related poison center reports between 2015 and 2025, with a marked acceleration beginning in 2023 that correlates temporally with the commercial emergence of concentrated semisynthetic 7-hydroxymitrgynine (7-OH) products (Eleanor Blair Towers et al., 2026).

Kratom users in the United States do not use kratom in isolation. A nationally representative survey of 1,842 US adults found that 71.4%–92% of respondents who used kratom also used other substances, and 42.9% had previously received addiction treatment (Covvey et al., 2020). Latent class analysis of 2019 National Survey on Drug Use and Health (NSDUH) data identified three distinct polysubstance use profiles among kratom users: a majority class using marijuana, alcohol, and tobacco concurrently; a second class adding psychedelic use; and a third class, comprising of 17.4% of kratom users, that also included heroin and prescription drug use (Falise et al., 2023). Notably, alcohol was present across all three classes. The population using kratom is, in other words, predominantly a polysubstance-using population already exposed to drugs whose interaction with kratom have not been systematically studied.

### The central problem

Adverse event data establish that kratom use is associated with serious harm, including death (Li et al., 2023; Eleanor Blair Towers et al., 2026). What these data cannot establish is whether kratom caused those deaths, contributed to them alongside other substances, or was present incidentally in individuals whose deaths were attributable to other causes. This distinction is foundational to any evidence-based regulatory or clinical response.

The scale of the polypharmacy problem is documented across multiple independent data sources. In the largest systematic case series, Corkery and colleagues analyzed 156 kratom-associated fatalities and found polysubstance involvement in 87%, with controlled and recreational drugs, therapeutic medications, or alcohol present (Corkery et al., 2019). A Nevada analysis found that mitragynine blood concentrations did not differ significantly between cases where kratom was listed as a contributing cause of death versus an incidental finding (Schmitt et al., 2021). This means that the same measured concentration could support either a causal or an incidental attribution depending on the medical examiner’s judgment, without a pharmacological threshold to anchor the determination (Striley et al., 2022). Pharmacovigilance analysis of 489 FAERS reports identified the 63-fold excess mortality signal, but as with all passive surveillance, this reflects association rather than causation (Li et al., 2023).

A further infrastructural problem compounds attribution: kratom alkaloids, including mitragynine and 7-OH, are not included in standard clinical or postmortem toxicology screens. The true number of deaths in which kratom was present is almost certainly undercounted. Conversely, some attributed kratom deaths may reflect cases where it was the only substance tested by specialized methods while other co-substances went unscreened. A further selection effect is relevant: a subset of kratom users specifically choose kratom because its alkaloids do not appear on standard immunoassay drug screens, enriching kratom-associated fatality populations for individuals actively managing drug use to avoid detection (Corkery et al., 2019).

Establishing causal contribution in a polypharmacy context would require, at minimum: a pharmacologically validated lethal concentration range for mitragynine and 7-OH in humans; an understanding of how this lethal concentration range changes as a function of tolerance; controlled data on how kratom alkaloids interact with other drugs in a polypharmacy context; and a forensic framework capable of distinguishing kratom-attributable from co-substance-attributable respiratory depression or organ failure. None of these currently exist.

### Scope of this review

This Scoping review maps the available evidence on kratom’s role in polypharmacy-associated adverse outcomes, following PRISMA extension for scoping reviews (PRISMA-ScR) guidance. We do not attempt a systematic synthesis or meta-analysis of outcomes data, nor do we adjudicate causal attribution in individual cases. Instead, we characterize what the literature can and cannot support and define the data gaps that must be addressed before causal conclusions are possible.

The review addresses four questions:

- What does the fatality and adverse event literature establish about the frequency and pattern of polysubstance use in kratom-associated deaths?
- What pharmacological mechanisms have been identified through which kratom could plausibly contribute to harm in a polypharmacy context?
- What are the forensic and methodological barriers to causal attribution in kratom-associated deaths?
- How does the emergence of concentrated semisynthetic 7-OH products alter the risk profile relative to traditional kratom preparations?

Sources included peer-reviewed original research, case reports and series, narrative reviews, pharmacovigilance analyses, and publicly available surveillance data from the FDA, CDC, and state health departments. Literature was identified through systematic PubMed searches supplemented by citation tracking and targeted grey literature searches through April 2026. Sources were evaluated for methodological quality and conflicts of interest.

### Regulatory context

Kratom occupies an unusual position in the US regulatory landscape. It is not scheduled under the Controlled Substances Act, not approved as a drug by the FDA to diagnose, cure, mitigate, prevent or treat any disease, not approved or recognized by the FDA as a dietary supplement, yet sold openly across most of the country. The FDA has stated that kratom cannot be lawfully added to conventional foods (FDA, 2025), but enforcement has been limited and inconsistent. In August 2016, the DEA published notice of its intent to place mitragynine and 7-OH in Schedule I on an emergency basis. Following an exceptional public response, including letters from members of Congress citing the need for further research on kratom as a potential treatment for opioid withdrawal, the DEA withdrew the notice (Prozialeck et al., 2019). That withdrawal was not a scientific finding that kratom is safe; it was a procedural response to public and political pressure, with the explicit expectation that the research base would be developed in the intervening period. A decade later, the evidence base has grown substantially, but the fundamental data gaps that prevented causal conclusions in 2016 remain unresolved, and the market has changed dramatically.

State-level responses have been fragmented and largely ineffective. Neither outright bans nor Kratom Consumer Protection Acts (KCPAs) have demonstrably reduced use. Nationally representative survey data found no statistically significant difference in past-year kratom use across banned states, KCPA states, and states with no policy, with online availability identified as the principal confounding factor that renders geographically bounded regulation structurally limited (Ellis et al., 2024). At the retail level, 46.2% of San Diego retailers were selling kratom 3 years after a citywide ban took effect (Bowdring et al., 2023). Meanwhile, the products being sold have become substantially more potent and pharmacologically distinct over time.

## Materials and methods

This scoping review was conducted following the Preferred Reporting Items for Systematic Reviews and Meta-Analyses extension for Scoping Reviews (PRISMA-ScR) guidance. A systematic literature search was conducted using the PubMed Advanced Search Builder (National Center for Biotechnology Information, Bethesda, MD, USA) through April 2026. Fourteen discrete search queries were executed to capture the breadth of relevant literature across pharmacological, epidemiological, clinical, and regulatory domains. Search items and result yields were as follows: kratom AND polysubstance (n = 23); kratom AND health effects AND public health (n = 122); kratom AND health effects AND polypharmacy (n = 2); kratom AND regulation (n = 87); kratom AND policy (n = 52); kratom AND mitragynine AND alkaloid (n = 386); kratom AND 7-hydroxymitragynine AND alkaloid (n = 133); kratom AND use patterns AND withdrawals NOT review (n = 69); kratom AND mitragynine AND CYP2D6 AND inhibitor (n = 6); CYP3A4 AND time-dependent inactivation (n = 254); kratom AND mitragynine AND CYP3A4 AND inhibitor (n = 9); CYP2D6 AND opioids AND SSRIs AND metabolism (n = 39); CYP2D6 AND polymorphism AND risk (n = 810); and human AND kratom AND polypharmacy (n = 1) (Figure 1). Database results were supplemented by citation tracking, reference list review, and targeted searches for grey literature including government surveillance reports, public health advisories, and regulatory documents from federal and state agencies.

**FIGURE 1:**
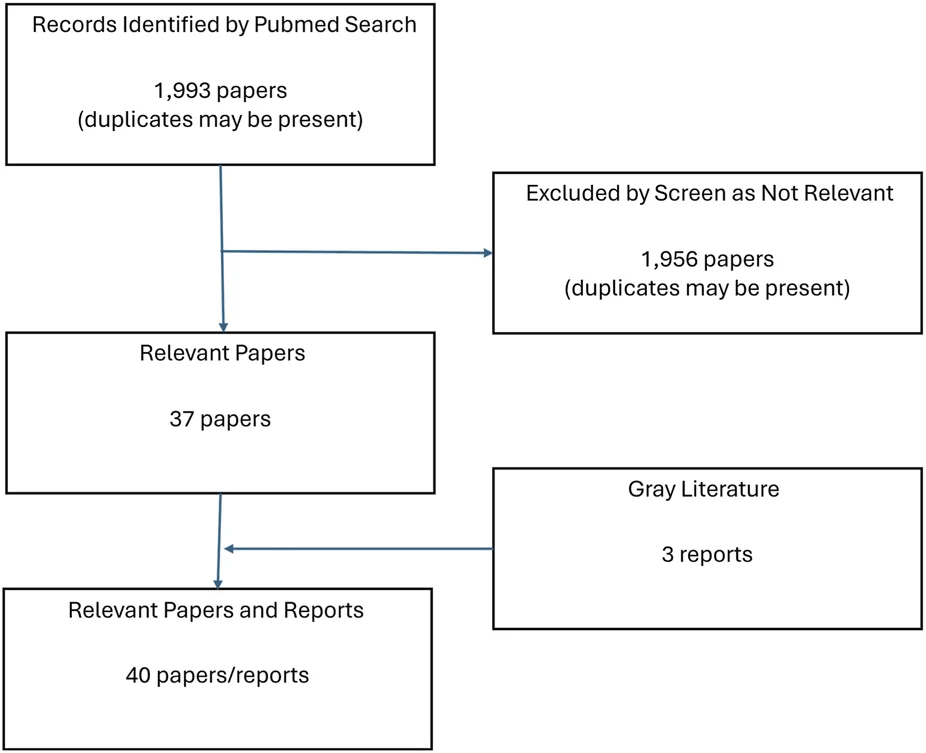
Flowchart depicting the number of papers and gray literature identified, the amount excluded as not relevant, and the final number of relevant papers/reports.

Sources were screened for relevance to kratom pharmacology, polypharmacy risk, fatality data, forensic toxicology, and regulatory policy. Consistent with PRISMA-ScR guidance, sources were not excluded on methodological quality grounds; instead, methodological limitations and conflicts of interest are characterized within the review narrative where relevant. Included source types encompasses peer-reviewed original research, case reports, case series, narrative reviews, pharmacovigilance analyses, and publicly available surveillance data.

Artificial intelligence writing assistance (Claude, Anthropic, San Francisco, CA, USA) was used during the drafting and editorial revision of this manuscript. All AI-assisted content was reviewed, verified against primary sources, and approved by the authors. Responsibility for the accuracy and integrity of the content rests solely with the authors.

## Review of the literature: kratom pharmacology

### Alkaloid composition and abundance

Kratom leaves contain more than 40 identified alkaloids. Mitragynine (MG) constitutes approximately 60%–66% of total alkaloid content and is the primary alkaloid in virtually all commercial preparations (Zhou et al., 2021). The remaining alkaloid fraction includes speciociliatine, speciogynine, paynantheine, and corynantheidine as major constituents, alongside minor alkaloids present at less than 1% of total content. Within this 1% fraction is 7-OH, the oxidized active metabolite of mitragynine that has emerged as the pharmacologically dominant compound in concentrated semisynthetic preparations (Zhou et al., 2021).

#### Mitragynine

Mitragynine is an indole alkaloid structurally distinct from classical opioids (Figure 2), lacking the phenol group characteristic of morphine and its derivatives, but acts at the same target. It binds the orthosteric site of the μ-opioid receptor (MOP), forming a conserved salt bridge with Asp147 characteristic of opioid receptor agonists and antagonists across drug classes (Zhou et al., 2021). At the MOP, mitragynine functions as a partial agonist with lower affinity than morphine.

**FIGURE 2:**
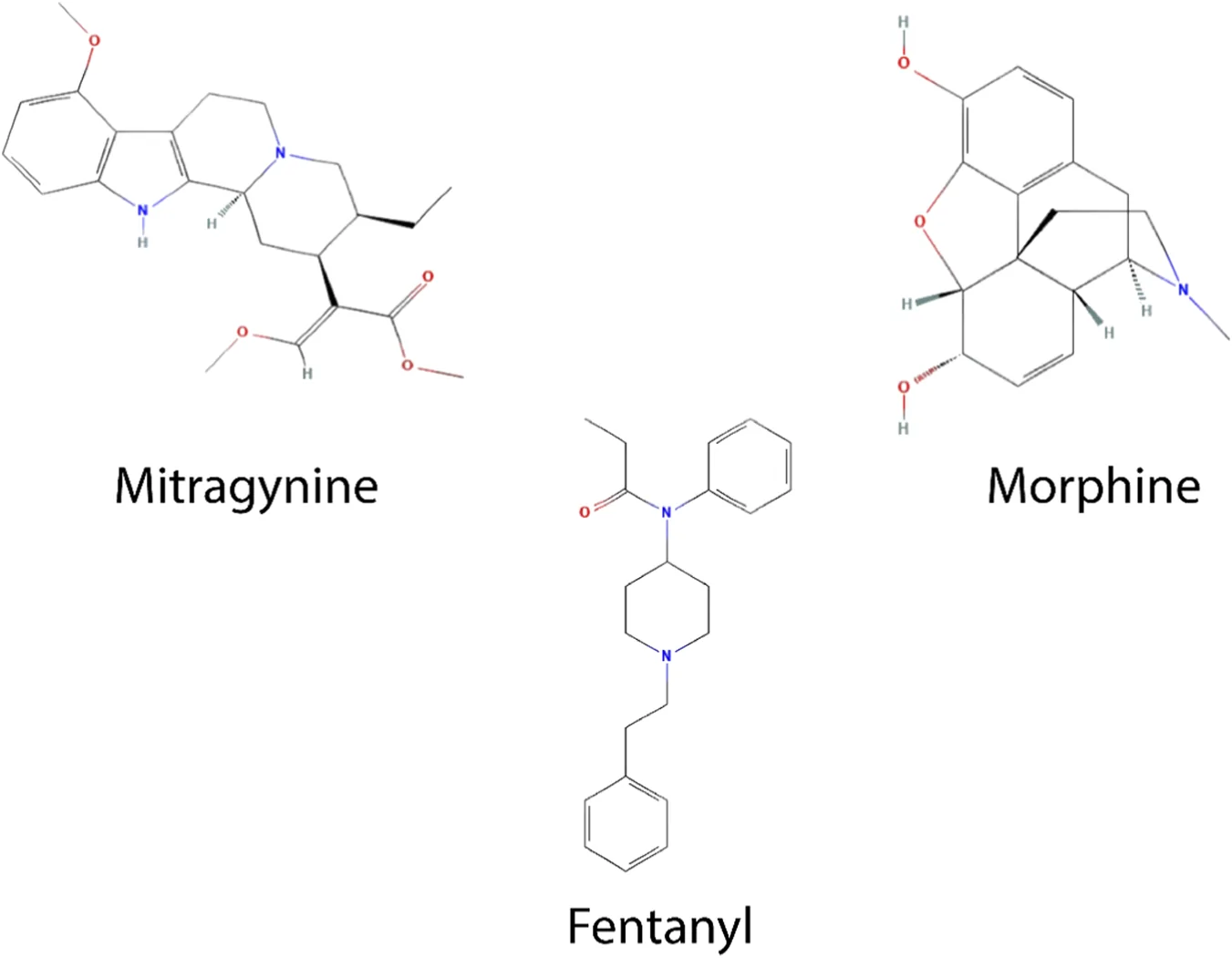
The structures of the μ-opioid receptor ligands mitragynine, morphine, fentanyl are depicted.

Mitragynine’s pharmacological activity extends beyond the opioid receptor to include adrenergic receptors, serotonergic receptors, signaling pathways, as well as modulation of the descending monoaminergic system involved in pain processing (Suhaimi et al., 2016; León et al., 2021; Obeng et al., 2022). This multi-receptor profile is clinically significant: mitragynine’s pharmacodynamic interactions with co-administered drugs extend beyond opioid receptor overlap to include serotonergic, adrenergic, and dopaminergic drug classes, and the pharmacological effects of kratom are not fully characterized by opioid receptor activity alone (Hanapi et al., 2021; Obeng et al., 2022).

Critically, mitragynine is also a pharmacokinetic actor. It is a substrate of hepatic cytochrome P450 enzymes, primarily CYP3A4 and CYP2D6, and simultaneously inhibits those same enzymes.

#### 7-Hydroxymitragynine–the active metabolite

7-OH is formed from mitragynine through hepatic oxidation, primarily via CYP3A4. Despite constituting less than 1% of the alkaloid content of traditional kratom leaf, it is pharmacologically more potent than the other alkaloids. 7-OH demonstrates approximately 46-fold greater MOP affinity than mitragynine and 13-fold greater potency than morphine in animal pain assays (Zhou et al., 2021; Sempio et al., 2025). Like mitragynine, 7-OH is a G-protein partial agonist at the MOP, but its substantially higher receptor affinity means that even relatively small quantities generated from mitragynine metabolism can drive significant opioid receptor-mediated effects. In human pharmacokinetic data from a controlled clinical trial, 7-OH plasma concentrations reached approximately one-fifth those of mitragynine; given the 46-fold potency advantage, 7-OH’s pharmacokinetic contribution is disproportionate to its measured concentration (Huestis et al., 2024; Sempio et al., 2025).

The prodrug relationship between mitragynine and 7-OH has important implications for polypharmacy risk. The effective opioid exposure from a given dose of kratom is not fixed, it depends on the rate of CYP3A4-mediated conversion of mitragynine to 7-OH, which varies substantially between individuals based on CYP3A4 expression level, concurrent medication, and metabolic state (Tanna et al., 2021). Additionally, mitragynine is a time-dependent inhibitor of CYP3A4, meaning inhibitory effect accumulates with repeated exposure rather than being concentration-dependent at a single time point (Tanna et al., 2021). This creates the potential for progressive impairment of CYP3A4-mediated clearance of co-administered substrates such as fentanyl, oxycodone, and alprazolam, with in-vitro-to-in vivo extrapolation predicting a clinically meaningful interaction magnitude. Clinical case reports are consistent with this potential interaction, but its magnitude and clinical significance have not been confirmed in controlled human pharmacokinetic studies (Brogdon et al., 2022; Vanani et al., 2023).

#### Product variability and dose unpredictability

The pharmacological picture above applies to preparations of known alkaloid composition. In practice, commercially available kratom products are neither standardized nor reliably disclosed. Analysis of 107 ready-to-drink kratom beverages found kratom content ranging from 20 to 500 mg per drink, with serving suggestions ranging from 1 to 15 servings per drink and inconsistent labeling that used “extract”, “alkaloids” and “mitragynine” interchangeably without standardized reference to alkaloid composition (Perry and Chin, 2025). To be clear, FDA has concluded that kratom is an unapproved new dietary ingredient, and that any dietary supplements or foods that are or contain kratom are adulterated and thus illegal (https://www.fda.gov/news-events/public-health-focus/fda-and-kratom; accessed 10 July 2026). The continued illegal marketing of kratom products at stores across the United States reflects an inability to fully enforce the law by the US FDA.

### Polypharmacy as the dominant pattern in kratom-associated deaths

#### Fatality literature across independent data sources

Across independent data sources spanning multiple surveillance systems, geographic populations, and time periods, polysubstance use is the dominant finding in kratom-associated deaths. Five sources converge on the same pattern (Table 1).

**TABLE 1:** Polysubstance involvement in kratom-associated fatality datasets.

Study	n Deaths	% Polydrug
(Olsen et al., 2019)	152	∼95.4
(Corkery et al., 2019)	156	87
(Eleanor Blair Towers et al., 2026)	233	79
(Smallets et al., 2025)	35	91.4
(Torrico et al., 2024)	4	100

The Olsen et al. data reflects unintentional overdose deaths specifically. Lower polydrug rates in larger, more recent surveillance datasets likely reflect inclusion of earlier years with less comprehensive toxicology panel coverage.

Across all five sources, the polydrug rate in kratom-associated deaths ranges from 79% to 100%. The upper bound of 100% reflects small sample size, while the lower figure likely underestimates true co-substance prevalence given historical gaps in detection. For the reasons described throughout this paper, we believe the 79%–100% rate to be highly uncertain, and that it does not accurately portray the prevalence of polydrug involvement in kratom-associated deaths.

#### Co-substances most commonly detected

Fentanyl and fentanyl analogs are the most commonly identified co-substance, detected in 65%–75% of kratom-associated deaths where co-substance data are reported (Olsen et al., 2019; Torrico et al., 2024). Heroin was detected in approximately 32.9% of cases in the SUDORS dataset (Olsen et al., 2019). Benzodiazepines were present in 15%–22.4% of cases across multiple studies; prescription opioids in 16%–19.7%; ethanol in 12.5%–22%; cocaine in 18.4%; antidepressants in approximately 14%; and cannabis in approximately 12% (Olsen et al., 2019; Eleanor Blair Towers et al., 2026). Frequencies are not mutually exclusive; multiple co-substances were present in many individual cases.

These findings are summarized in Table 2.

**TABLE 2:** Substances most frequently co-detected with kratom in polypharmacy death investigations.

Rank	Substance	Frequency	Source
1	Fentanyl/fentanyl analogs	65.1%–75%	(Olsen et al., 2019; Torrico et al., 2024)
2	Heroin	32.9%	(Olsen et al., 2019)
3	Ethanol	12.5%–22%	(Olsen et al., 2019; Eleanor Blair Towers et al., 2026)
4	Benzodiazepines	15%–22.4%	Multiple studies
5	Prescription opioids	16%–19.7%	(Olsen et al., 2019; Eleanor Blair Towers et al., 2026)
6	Cocaine	18.4%	(Olsen et al., 2019)
7	Antidepressants	14%	(Eleanor Blair Towers et al., 2026)
8	Cannabis	12%	(Eleanor Blair Towers et al., 2026)

Frequencies are not mutually exclusive; multiple co-substances were present in many individual cases. The Olsen et al. data reflects SUDORS, surveillance of unintentional overdose deaths across 27 states.

#### What the fatality literature can and cannot establish

These data establish that polysubstance use is the dominant context in which kratom-associated deaths occur, meaning any regulatory or clinical response that fails to account for polypharmacy is responding to an incomplete picture.

What these data cannot establish is the causal contribution of kratom to any individual death. A death in which both fentanyl and mitragynine are detected may represent a case in which fentanyl was solely responsible, one in which kratom pharmacokinetically amplified fentanyl toxicity to a lethal level, or one in which both independently contributed. The existing data cannot reliably distinguish between these possibilities. This causal ambiguity is compounded in the kratom context by the absence of validated human lethal concentration thresholds, the inconsistency of kratom detected in standard toxicology panels, and the pharmacological complexity of kratom’s interactions with co-substances.

Analysis of postmortem mitragynine concentrations across fatal and non-fatal cases showed substantial overlap: mitragynine concentrations in postmortem cases (541) ranged from 5.4 to 11,000 ng/mL (mean 360, median 120 ng/mL), while concentrations in surviving individuals ranged from 8.8 to 1,000 ng/mL (mean 133, median 62 ng/mL), with a substantial overlap zone in which concentration alone cannot distinguish fatal from non-fatal exposure (Papsun et al., 2023). The only analytically confirmed sole-substance kratom fatality in the literature involved a central blood mitragynine concentration of 7,500 ng/mL alongside significant underlying cardiovascular and hepatic pathology (Mata and Chang, 2023). This case provides evidence consistent with the possibility of a fatal outcome involving kratom in the absence of detected co-substances; however, the contribution of the underlying cardiovascular and hepatic pathology prevents unambiguous attribution to kratom alone.

### Mechanisms by which kratom may contribute to polypharmacy deaths

Two pharmacological pathways provide a mechanistically coherent framework for understanding how kratom alkaloids could augment the lethality of co-administered substances. These pathways operate through distinct but potentially overlapping mechanisms. Each is supported by a combination of *in vitro* mechanistic data, preclinical evidence, and human case documentation. None have been evaluated in a controlled prospective human drug interaction study.

#### Pathway 1: additive and synergistic CNS and respiratory depression via μ -opioid receptor overlap

Mitragynine and 7-OH are partial agonists at the MOP, the primary receptor mediating opioid-induced respiratory depression (Zhou et al., 2021). 7-OH demonstrates 13-fold greater MOP potency than morphine in animal models, and both alkaloids activate G-protein-mediated intracellular signaling shared with classical opioid agonists (Zhou et al., 2021; Sempio et al., 2025). Based on the law of mass action, when kratom alkaloids are co-administered with full MOP agonists such as fentanyl, heroin, oxycodone, or hydromorphone, the combined receptor occupancy and downstream signaling may exceed what either agent produces alone. The magnitude and direction of this interaction depends on the free concentrations, fractional occupancies, receptor affinities, and intrinsic efficacies of the competing ligands, as well as receptor reserve and downstream signal amplification (Olsen et al., 2019; Torrico et al., 2024).

Wang and Walker (2018) reported a fatal case in which oxycodone at 0.19 mg/L was combined with therapeutic concentrations of lorazepam and mitragynine at 2,500 ng/mL, in a case involving fatal CNS depression and bronchopneumonia. It is important to note that 0.19 mg/L of oxycodone is within the lethal range for some users. Shi and Shea (2024) documented fatal combined toxicity of hydromorphone and kratom (Shi and Shea, 2024). In both cases, it is difficult to attribute death to the opioid drug, kratom, or the mixture without additional information on the people involved.

#### Pathway 2: CYP enzyme inhibition and pharmacokinetic drug interaction

The second pathway operates at the level of drug metabolism rather than receptor pharmacodynamics. Mitragynine is a time-dependent inhibitor of CYP3A4 and competitive inhibitor of CYP2D6, with additional inhibitory activity at CYP2C9 and CYP1A2 (Kamble et al., 2020; Tanna et al., 2021). These four isoforms are collectively responsible for the hepatic metabolism of a large portion of clinically used drugs. *In vitro* studies indicate that inhibition of these enzymes could increase plasma concentrations of susceptible co-administered substrates, potentially increasing otherwise therapeutic or sub-toxic exposures to concentrations associated with greater toxicity (Table 3).

**TABLE 3:** CYP enzymes inhibited by kratom alkaloids and key substrates at risk.

CYP enzyme	Kratom effect	Key substrates at risk
CYP3A4	Tim-dependent inhibition	Fentanyl, oxycodone, quetiapine, alprazolam, methadone
CYP2D6	Reversible Inhibition	Venlafaxine, fluoxetine, oxycodone, tramadol, codeine
CYP2C9	Inhibition	Warfarin, ibuprofen, phenytoin
CYP1A2	Inhibition	Clozapine, duloxetine, theophylline

The time-dependent nature of CYP3A4 inhibition is particularly significant: the inhibitory effect accumulates with repeated exposure rather than being concentration-dependent at a single time point (Tanna et al., 2021). A kratom user who begins with occasional use may experience limited CYP3A4 inhibition, but a chronic daily user at steady-state mitragynine plasma concentrations may be substantially impairing CYP3A4 function without awareness. For a user also taking fentanyl, oxycodone, or another CYP3A4-dependent opioid, this progressive enzyme inhibition could increase opioid plasma concentrations over time; however, the magnitude and clinical significance of this interaction have not been established in controlled human studies.

CYP2D6 inhibition adds pharmacogenomic complexity. CYP2D6 is among the most polymorphic drug-metabolizing enzymes in the human genome, with allele frequencies producing clinically distinct metabolizer phenotypes that vary substantially across racial and ethnic populations. Poor metabolizers are already operating with reduced CYP2D6 function and may be at higher baseline risk from kratom-mediated CYP2D6 interactions. Concurrently, ultrarapid metabolizers may be partially buffered. The clinical consequence is that the same kratom dose and the same co-substance dose can produce dramatically different plasma concentration profiles in different individuals.

Vanani and colleagues (2023) reported a patient who had taken amitriptyline, a CYP2D6 substrate, without incident for 15 years, and developed progressive anticholinergic toxicity after adding kratom at escalating doses. These symptoms resolved completely on kratom cessation, consistent with CYP2D6 inhibition reversal. Brogdon and colleagues (2022) reported a more complex case where ∼90 g daily kratom use combined with venlafaxine 150 mg and quetiapine 300 mg resulted in the patient developing serotonin syndrome meeting Hunter Criteria and findings consistent with quetiapine toxicity. The authors proposed a potential pharmacokinetic interaction involving CYP3A4 inhibition. A fatal case reported by McIntyre and colleagues (2015) is particularly instructive: all co-substances present (venlafaxine, diphenhydramine, and mirtazapine) were measured at concentrations consistent with therapeutic rather than toxic exposures, yet the case resulted in a fatal outcome. One potential explanation is CYP-mediated amplification of the combined serotonergic drug burden to a toxic threshold.

### The forensic attribution problem

The two mechanistic pathways described above sharpen rather than resolve the forensic attribution problem. If kratom contributes through pharmacokinetic amplification of co-substance concentrations or additive receptor-level effects, concentration-based forensic approaches alone may be insufficient to identify or quantify that contribution. Three specific limitations compound this challenge.

#### Absence of a validated human lethal concentration range

Medical examiners can rely on established toxic and lethal reference ranges for ethanol, many prescription opioids, and numerous other drugs of misuse. No equivalent validated reference range exists for mitragynine or 7-OH. Postmortem mitragynine concentrations overlap substantially between fatal and non-fatal cases, precluding concentration-based determinations of causal contributions (Papsun et al., 2023). The only analytically confirmed sole-substance kratom fatality in the literature, a central blood mitragynine of 7,500 ng/mL with significant underlying cardiovascular and hepatic pathology, cannot serve as a validated forensic threshold (Mata and Chang, 2023).

#### Postmortem redistribution (PMR) of mitragynine

Drug concentrations in postmortem blood are affected by redistribution from tissues into blood after death. Initially, PMR was characterized only from isolated case reports: a central-to-peripheral blood ratio of 0.83 (McIntyre et al., 2015) and 2.27 in another (Mata and Chang, 2023), a nearly three-fold spread that precluded meaningful cross-case comparisons. However, Osawa and Johnson (2025) reported a systematic multi-case PMR dataset, analyzing central and peripheral blood from 51 postmortem cases. The average heart blood-to-femoral blood ratio was 1.37 for mitragynine and 1.08 for 7-OH, suggesting a mild but largely uncertain difference between central and peripheral concentrations. Notably, large average mitragynine-to-7-OH ratios were observed across matrices: 30.9 in femoral blood, 32.4 in heart blood, 14.8 in vitreous humor, and 16.9 in urine, with the consistently lower ratios in vitreous humor and urine suggesting those matrices may better reflect antemortem pharmacological exposure. Without validated PMR correction factors, cardiac blood mitragynine concentrations reported in surveillance studies cannot be reliably compared to perimortem concentrations or to each other across cases. Furthermore, care must be taken when comparing femoral and cardiac blood concentrations of mytragynine and 7-OH in postmortem cases.

#### Instability of 7-OH in postmortem specimens

Osawa and Johnson (2025) directly quantified the pre-analytical stability of both alkaloids in postmortem matrix under refrigerated conditions. Mitragynine was stable for up to 30 days at 4 °C with less than 20% analyte loss. By contrast, 7-OH was stable for only 7 days under the same conditions, with concentrations falling more than 50% from initial values by 60 days of refrigerated storage. Methanolic preparations of both compounds were stable for 3 months at −20 °C, suggesting that extraction and storage conditions are a significant and modifiable source of analytical error (Osawa and Johnson, 2025).

Cases in which blood was stored under comparable refrigerated conditions for more than 7 days before analysis may underestimate 7-OH concentrations present at specimen collection. Given that 7-OH carries approximately 46-fold greater μ-opioid receptor affinity than mitragynine, even a partial loss of 7-OH signal represents a disproportionate underestimation of the opioid receptor-mediated pharmacological burden at the time of death.

Taken together, these limitations create bidirectional uncertainty: the prevalence of kratom exposure in polypharmacy deaths may be underestimated in surveillance data because of incomplete testing and analytical limitations, while its causal contribution may be overstated in individual cases when attribution is made without validated concentration thresholds. Resolving this bidirectional uncertainty requires systematic forensic reference data.

### The emerging 7-OH semisynthetic risk

#### A pharmacologically distinct product class

Traditional dried kratom leaf and tea preparations contain 7-OH at less than 2% of total alkaloid content. The emergence since approximately 2023 of concentrated semisynthetic 7-OH products–gummies, tincture, sublingual tablets, nasal sprays–represents a qualitative shift in the risk landscape that cannot be adequately characterized within a botanical supplement framework (Alsbrook et al., 2025; Smith et al., 2025).

Semisynthetic 7-OH is produced through chemical modification of mitragynine extracted from the kratom leaf, yielding concentrated 7-OH at purities and doses bearing no relationship to the alkaloid profile of natural kratom preparations. Where a standard kratom preparation might deliver trace quantities of 7-OH as a fraction of total alkaloid content, semisynthetic products have been documented delivering 7-OH as the primary active ingredient at doses (milligram doses) much higher than consuming the natural leaf (Smith et al., 2025).

#### Pharmacological profile and opioid equivalence

7-OH demonstrates nanomolar μ-opioid receptor binding affinity, and preclinical data confirm dose-dependent respiratory depression, tolerance development, physical dependence, and an opioid-type withdrawal syndrome (Alsbrook et al., 2025). These are the defining pharmacological characteristics of controlled opioid analgesics, not dietary supplements. The G-protein bias of kratom alkaloids does not eliminate respiratory depression risk at concentrations achievable with concentrated semisynthetic preparations. Instead, it shifts the dose-response curve in a manner that has not been characterized specifically for semisynthetic 7-OH and cannot be assumed to confer the same partial protection against respiratory depression that may exist at lower concentrations associated with traditional use.

Concentrated semisynthetic 7-OH presents a substantially different pharmacological hazard profile from traditional kratom preparations. Its greater MOP potency and much higher 7-OH exposure provide a mechanistic basis for concern about increased polypharmacy risk. However, comparative human data are not available to establish whether, or by how much, its polypharmacy fatality risk exceeds that of traditional kratom. When 7-OH is combined with other CNS depressants such as benzodiazepines, alcohol, or gabapentin, the receptor-level interactions described in Pathway 1 provide a plausible mechanism for increased respiratory depression. CYP inhibition mediated by the mitragynine content of traditional kratom may be attenuated or absent in semisynthetic 7-OH products containing little or no mitragynine; consequently, the mechanisms of potential polypharmacy toxicity may differ between these product classes.

#### Clinical and surveillance evidence

Clinical and public health evidence for the acute toxicity of concentrated 7-OH products has emerged rapidly since 2023. Pullman and colleagues (2026) reported cardiopulmonary arrest in a 29-year-old who consumed 190 mg of 7-OH while intoxicated with alcohol, requiring 10 min of CPR and reversal with two intravenous doses of naloxone. The prompt and complete response to naloxone strongly supports MOP-mediated respiratory depression as a mechanism underlying the clinical presentation. Los Angeles County public health authorities confirmed three fatalities linked to concentrated 7-OH products, representing among the first documented jurisdiction-level fatality data specifically attributable to semisynthetic 7-OH (http://publichealth.lacounty.gov/phcommon/public/media/mediapubhpdetail.cfm?prid=5139). The Texas Poison Center Network documented 19 cases in 2025 specifically involving concentrated 7-OH, of which 11 required medical treatment (https://www.dshs.texas.gov/news-alerts/serious-illnesses-associated-7-oh-use). At the national level, the post-2023 acceleration in NPDS kratom-related reports correlates temporally with the commercial emergence of concentrated 7-OH products. Because NPDS kratom reports do not reliably distinguish traditional kratom from concentrated 7-OH products, these surveillance data cannot determine whether 7-OH contributed to the observed increase (Eleanor Blair Towers et al., 2026).

A near-fatal case reported by Sharma and colleagues (2025) illustrates the clinical complexity of concentrated 7-OH interactions with opioid treatment medications. A patient using 700–800 mg/day of 7-OH extract presented with precipitated withdrawal from buprenorphine, required intubation and ICU-level care, and had a standard immunoassay toxicology screen negative for all opioids. This case illustrates two compounding problems: concentrated 7-OH products create clinical presentations that emergency and addiction medicine providers are not prepared to recognize or manage, and the forensic and clinical detection infrastructure for opioids does not extend to kratom alkaloids even in clinical presentations consistent with opioid-mediated toxicity.

#### The MGM-15 and MGM-16 horizon

Beyond 7-OH, a further tier of synthetic and semisynthetic kratom derivatives has begun to appear in consumer markets. MGM-15 and MGM-16 are semisynthetic indoloquinolizine opioids developed from 7-OH as part of medicinal chemistry programs targeting orally active dual μ- and δ-opioid receptor agonists (Matsumoto et al., 2014). MGM-16 carries a 9-fluoro substituent; MGM-15 is the non-fluorinated analogue. In preclinical models, MGM-16 demonstrates antinociceptive potency up to 71-fold greater than morphine by subcutaneous dosing and over 200-fold greater after oral administration (Matsumoto et al., 2014). By 2025, MGM-15 had appeared in US online vendor listings as a research chemical or reference material, frequently categorized under “7-hydroxymitragynine” product listings in a terminological conflation that obscures the pharmacological distinction (Alsbrook et al., 2025). The polypharmacy fatality risk of MGM-15 and MGM-16 has not been characterized in human populations. Their high opioid-receptor potency nevertheless provides a strong pharmacological basis for concern regarding serious opioid-mediated toxicity, particularly when combined with other CNS depressants. Their emergence under kratom product categorization means that surveillance systems designed to track kratom-associated deaths will misclassify MGM-15 and MGM-16 exposures unless toxicology panels are expanded to include these compounds.

## Data gaps and research priorities

Six research priorities must be addressed before the causal contributions of kratom and 7-OH’s to polypharmacy deaths can be assessed reliably.

Gap 1: Lack of established human toxic and lethal concentration reference ranges for mitragynine or 7-OH. Data should be multicenter, stratified by specimen matrix, co-substances and their concentrations, tolerance, product type, and underlying pathology.

Gap 2: Lack of validated postmortem redistribution reference dataset for mitragynine and 7-OH. The community requires independent replication and formal validation of postmortem redistribution correction factors that can be applied reliably across casework. This should include data across central and peripheral blood, vitreous humor, urine, and tissue matrices from the same cadavers.

Gap 3: Lack of standardized specimen-handling protocols for 7-OH in postmortem specimens. Collection, storage, extraction, stability limits, and reporting requirements need to be standardized.

Gap 4: There is a need for prospective pharmacokinetic interaction studies. These should focus on the highest-priority interaction studies, based on co-substance frequency data and mechanistic profile, such as mitragynine combined with fentanyl or oxycodone; mitragynine combined with venlafaxine or fluoxetine; and mitragynine combined with alcohol.

Gap 5: Lack of pharmacogenomic data in kratom-associated fatality or adverse event datasets. We need better insights into the population level distribution of CYP2D6-related risk and the contribution of pharmacogenomic variation to individual adverse outcomes.

Gap 6: Need improved surveillance infrastructure distinguishing traditional kratom from semisynthetic 7-OH products. We need to distinguish between botanical kratom, conventional extracts, concentrated 7-OH products, and synthetic or semisynthetic derivatives such as MGM-15 and MGM-16.

## Conclusion

Kratom is not a pharmacologically simple substance, and the public health question it poses is not a simple one. There are still far more research questions that need to be addressed to understand the polypharmacy impacts of kratom alkaloids.

Polysubstance use is the dominant context in which kratom-associated deaths occur. It has been documented consistently across independent surveillance systems, geographic populations, and time periods at rates around 80%. Two pharmacologically coherent mechanisms provide a plausible and documented basis for kratom’s potential role in polypharmacy deaths: μ-opioid receptor-mediated CNS depression and CYP enzyme-mediated pharmacokinetic amplification of co-substance toxicity. These mechanisms establish biological plausibility but do not establish causal contribution to individual detahs. Determining whether, and under what circumstances, these mechanisms contribute to fatal outcomes requires further investigation.

The data gaps preventing causal confirmation are fundamental and have persisted for a decade without resolution. The absence of validated human lethal concentration thresholds, the absence of postmortem redistribution reference data, the instability of 7-OH in forensic specimens, and the absence of controlled human pharmacokinetic interaction data collectively mean that medical examiners, clinicians, and regulators are making consequential decisions about kratom without the evidentiary foundation required. Closing these gaps is a prerequisite for evidence-based kratom policy.

The emergence of concentrated semisynthetic 7-OH products and their synthetic derivatives since 2023 have fundamentally altered the risk landscape in ways that existing regulatory frameworks are not equipped to address. These products possess pharmacological characteristics associated with potent opioids: they produce μ-opioid receptor-mediated respiratory depression, tolerance, physical dependence, and withdrawal, yet are sold without prescription, standardized dosing information, or any requirement that purchasers be informed of their opioid character. However, their polypharmacy fatality risk remains unknown, similar to traditional kratom preparations.

The evidence is not yet sufficient to answer causal questions focused on kratom polypharmacy.

## Future directions

Future work should focus on addressing the six data gaps. In addition, human relevant *in vitro* and computational studies are required to better understand the pharmacodynamics of μ-opioid receptor (partial) agonism, and g-protein vs. beta-arrestin bias, especially in the polypharmacy context. It should be noted that the pharmacodynamics issues are not specific to polypharmacy, which is why we did not list them as a data gap; however, it should be recognized that the current literature is still not clear about the pharmacodynamics of mitragynine or 7-OH.
